# Black Soldier Fly (*Hermetia illucens*) Larvae Protein Derivatives: Potential to Promote Animal Health

**DOI:** 10.3390/ani10060941

**Published:** 2020-05-29

**Authors:** Ange Mouithys-Mickalad, Eric Schmitt, Monika Dalim, Thierry Franck, Nuria Martin Tome, Michel van Spankeren, Didier Serteyn, Aman Paul

**Affiliations:** 1 Centre of Oxygen, Research and Development—University of Liege, 4000 Liège, Belgium; amouithys@uliege.be (A.M.-M.); t.franck@ulg.ac.be (T.F.); didier.serteyn@ulg.ac.be (D.S.); 2 Protix B.V., 5107 NC Dongen, The Netherlands; eric.schmitt@protix.eu (E.S.); monika.dalim@protix.eu (M.D.); nuria.tome@protix.eu (N.M.T.); michel.vanspankeren@protix.eu (M.v.S.)

**Keywords:** *Hermetia illucens*, chickenmeal, fishmeal, proteins, DPPH, ABTS, myeloperoxidase, neutrophil response, immune response, antioxidant activity

## Abstract

**Simple Summary:**

In European countries, black soldier fly larvae (BSF) proteins are gaining rapid acceptance as high value protein ingredients in pet food and aquaculture feed formulations. BSF protein derivatives (proteins and protein hydrolysates) contain a significant share of short-chain peptides that are known to possess antioxidant behavior. In the present study, the *in vitro* antioxidant potential of BSF protein derivatives was analyzed using five different models. Chickenmeal and fishmeal are commonly used in pet food and aquaculture feed formulations and hence were used as industrial benchmarks. The results obtained during this study show that chickenmeal and fishmeal offer little or no advantage in protecting animal cells against the oxidative damage resulting from neutrophils and myeloperoxidase response. Moreover, chickenmeal and fishmeal even show pro-oxidant responses in some of the models tested during this study. It was found that the BSF protein derivatives used in this study could be effective in protecting the animal cells from oxidative damage as a consequence of immune response.

**Abstract:**

European legislation permits the inclusion of insect proteins in pet and aquaculture diets. Black soldier fly larvae (BSF) are one of the most actively produced species due to their low environmental impact and nutritional characteristics. BSF protein derivatives (proteins and protein hydrolysates) contain a substantial amount of low molecular weight peptides that are known to possess antioxidant potential. In this study, the *in vitro* antioxidant potential of commercial BSF proteins and protein hydrolysates was investigated for (1) radical scavenging activity, (2) myeloperoxidase activity modulation, and (3) neutrophil response modulation. Chickenmeal and fishmeal are commonly used in pet food and aquaculture formulations. Hence, both were used as industrial benchmarks during this study. The results indicate that fishmeal and chickenmeal are ineffective at suppressing the oxidative damage caused by neutrophil response and myeloperoxidase activity. Fishmeal and chickenmeal even exhibit pro-oxidant behavior in some of the models used during this study. On the other hand, it was found that BSF protein derivatives could be effective in protecting against the cellular damage resulting from neutrophil and myeloperoxidase activities. The outcomes of this study indicate that BSF protein derivatives could be potentially included in pet food and aquaculture feed formulations as health-promoting ingredients.

## 1. Introduction

Insects are commonly consumed as food in many cultures around the world [1,2,3]. In European countries, insect proteins are gaining rapid acceptance as high value protein ingredients in animal diets. The European Union has already approved the inclusion of insect proteins in pet food and aquaculture feed formulations [4]. Chickenmeal and fishmeal are common ingredients in pet food and aquaculture feed preparations, respectively [5,6]. Insect proteins are increasingly being viewed as an alternative to chickenmeal and fishmeal in these markets [4]. Amongst all the insect being produced on industrial scale, the black soldier fly (*Hermetia illucens*) larvae has gained special attention due to its ability to grow on a wide range of organic residues and unique nutritional composition [7,8]. The nutritional suitability of black soldier fly larvae (BSF) proteins in aquaculture and pet diets is well established [9,10,11,12,13].

Pets develop a wide range of health disorders with age. Aging can accelerate the free radical damage in a pet’s body, which might lead to cognitive and locomotor system malfunctioning [14]. Similarly, oxidative stress in fish as a result of immune response could lead to compromised health [15]. Neutrophils (white blood cells) are responsible for the primary defense mechanism of the body. Upon receiving the signal, neutrophils rush to the site of intrusion by pathogenic microbes. Then, neutrophils inactivate the pathogens by phagocytosis and the release of reactive oxygen species (ROS). The production of ROS is crucial for the host defense [16,17]. However, in the long term, excessive ROS production by neutrophils could damage animal cells and might lead to cellular aging, cancer, reduced immunity, etc. [18]. Dietary interventions that can scavenge ROS may help in reducing oxidative damage in the animal body and resulting health conditions [14].

Some short-chain peptides and free amino acids are known to possess antioxidant activity. These molecules can actively scavenge ROS and free radicals [19]. Studies on preparations obtained from the hydrolysis of *Amphiacusta annulipes*, *Bombyx mori*, *Gryllodes sigillatus*, *Locusta migratoria*, *Schistocerca gregaria*, *Tenebrio molitor,* and *Zophobas morio* proteins have indicated the strong antioxidant potential of insect protein hydrolysates [20,21,22,23]. Research institutes and companies are currently developing methods leading to the production of BSF protein hydrolysates that have superior nutritional properties [24,25,26,27]. BSF proteins hydrolysates have a significant share of proteins <1000 Da. This includes a mixture of short-chain peptides and free amino acids [24]. However, until now, only a few studies have been realized to evaluate the antioxidant potential of BSF protein hydrolysates. Firmansyah and Abduh [27] evaluated the DPPH (2,2-diphenyl-1-picrylhydrazyl) scavenging activity of a BSF protein hydrolysate. On the other hand, Zhu et al. [26] evaluated the DPPH, ABTS (2,2’-azino-bis(3-ethylbenzothiazoline-6-sulfonic acid), superoxide, and hydroxyl radical scavenging activity of BSF protein hydrolysates. No studies have been realized to date that evaluate the antioxidant activity of BSF protein hydrolysates using fundamental enzymatic and cellular models. Therefore, the antioxidant potential of BSF protein hydrolysates is poorly understood on a fundamental level. Detailed investigations on the *in vitro* antioxidant activity of BSF proteins and protein hydrolysates may unlock new applications of these protein derivatives to improve animal health.

The current study investigates the antioxidant potential of BSF proteins and protein hydrolysates, using (1) radical scavenging models involving DPPH and ABTS; (2) enzymatic models involving myeloperoxidase activity; and (3) a cellular model involving neutrophil response. Chickenmeal and fishmeal were used as industrial benchmarks in this study.

## 2. Materials and Methods

### 2.1. Reagents

All the reagents were of analytical grade. Dimethyl sulfoxide, methanol, ethanol, calcium chloride, potassium chloride, sodium chloride, hydrogen peroxide, and Tween-20 were purchased from Merck (VWR, Leuven, Belgium). Sodium nitrite, bovine serum albumin, phorbol 12-myristate 13-acetate, and Percoll^TM^ were purchased from Sigma (Bornem, Belgium). Aqueous extracts and solutions were made in Milli-Q water obtained using Milli-Q water system (Millipore, Bedford, MA, USA). Bicinchoninic acid and copper (II) sulfate solutions were purchased from Sigma (Steinheim, Germany). Whatman filter paper grade 4 (270 mm) was purchased from Amersham (Buckinghamshire, UK). A Sterlip 30 mL disposable vacuum filter system was purchased from Millipore (Bedford, MA, USA). 2,2-Diphenyl-1-picrylhydrazyl and 2’-azino-bis(3-ethylbenzthiazoline-6-sulfonic acid) were purchased from Aldrich (Darmstadt, Germany). 8-amino-5-chloro-7-phenylpyrido[3,4-d]pyridazine-1,4(2H,3H)dione (L-012) was purchased from Wako Chemicals (Neuss, Germany).

### 2.2. Raw Materials

Chickenmeal (CM) and fishmeal (FM) were purchased from an online webshop in September 2019. The chemical composition of both ingredients as declared by the supplier is indicated in Table 1.

BSF meat (BSF-P), BSF hydrolyzed meat (BSF-HP), and BSF aqueous protein hydrolysate (BSF-APH) were provided by Protix B.V. (Dongen, The Netherlands) in October 2019. According to the supplier, (1) BSF-P was pasteurized minced meat that was supplied frozen at −20 °C (brand name: PureeX^TM^). BSF-P is partially defatted and dried to produce BSF proteinmeal (brand name: ProteinX^TM^). (2) BSF-HP was enzymatically hydrolyzed and pasteurized minced meat, which was also supplied frozen at −20 °C (brand name: PureeX_pro_^TM^). (3) BSF-APH was the hydrolysate of water-soluble BSF proteins (brand name: ProteinAX_pro_^TM^). The details about each hydrolysis step (type of enzyme and hydrolysis conditions) employed for the production of BSF-HP and BSF-APH were not disclosed by the supplier. It was also indicated that BSF-APH has high solubility in water (>95%). The chemical composition of all three ingredients as declared by the supplier is indicated in Table 2.

Water-soluble extracts were prepared for CM, FM, BSF-P, and BSF-HP. These products (100 g each) were dissolved with six times volumes of Milli-Q water based on their respective dry matter contents (e.g., BSF-P had dry matter content of 33.3% and was diluted 200 mL Milli-Q water) and stirred for 2 h on a magnetic stirrer. Post centrifugation (1000× *g* for 30 min at 4 °C), the top fat layer was removed, and the supernatant was filtered using a Whatman filter (Grade 4). The centrifugation and filtration steps were repeated to remove all non-soluble residues. Finally, the supernatant was filtered using a Sterlip filter (50 mL, 0.22 µm) and freeze dried over a period of two days to obtain respective water-soluble extract powders. BSF-APH was used directly because it has water solubility >95%. All four water-soluble extract and BSF-APH powders were stored in a desiccator (at 18 °C) until further use.

### 2.3. Protein Quantification

The protein content of the four water-soluble extracts and BSF-APH powder was analyzed using bicinchoninic acid (BCA) protein assay [28]. The calibration curve was obtained using bovine serum albumin (BSA) as standard at concentrations of 0, 0.125, 0.25, 0.5, and 1 mg/mL. Stock solutions of 3 mg/mL water-soluble extracts and BSF-APH were used for analysis. A test solution was made by dissolving 4900 µL BCA (49/50) and 100 µL copper (II) sulfate (1/50). Sample stock solutions (10 µL) and test solution (200 µL) were added in wells of a 96-well plate. This plate was incubated at 37 °C for 30 min, and absorbance was measured at 450 nm using a Multiscan Ascent (Fisher Scientific, Asse, Belgium).

### 2.4. DPPH Assay

DPPH radical scavenging activity was analyzed according to the protocol of Brand-Willams et al. [29], with some modifications. DPPH test solution was made by dissolving 10.5 mg of DPPH in 40 mL of ethanol. Test solution was made fresh and stored in the dark until further use. DPPH working solution was made by diluting the test solution with 10 times ethanol (to obtain absorbance of 0.6 to 0.8 at 517 nm). DPPH working solution (1920 µL) was mixed with 20 µL of the sample dilutions (four water-soluble extracts and BSF-APH in Milli-Q water) to obtain a final concentration of 0.0125, 0.025, 0.05, 0.1, and 0.2 mg/mL. The decrease in absorbance after 30 min of incubation in the dark was recorded at 510 nm using an HP 8453 UV-vis spectrophotometer (Agilent Technologies, Waldbronn, Germany). Instead of sample dilutions, only Milli-Q water was used in case of control.

### 2.5. ABTS Assay

ABTS cation radical scavenging activity was analyzed according to the protocol of Arnao et al. [30], with some modifications. ABTS test solution was made by dissolving 7.0 mmol/L ABTS and 2.45 mmol/L potassium persulfate in Milli-Q water. The test solution was kept overnight in the dark at room temperature. ABTS working solution was made by diluting with methanol to obtain the absorbance between 0.7 and 0.8 at 734 nm. ABTS working solution (1920 µL) was mixed with 20 µL of samples dilutions (four water-soluble extracts and BSF-APH in Milli-Q water) to obtain final concentrations of 0.0125, 0.025, 0.05, 0.1 and 0.2 mg/mL. The decrease in absorbance after 30 min of incubation in dark was recorded at 734 nm using an HP 8453 UV-vis spectrophotometer (Agilent Technologies, Waldbronn, Germany). Instead of sample dilutions, only Milli-Q water was used in case of the control.

### 2.6. Myeloperoxidase (MPO) Activity Using Specific Immunological Extraction Followed by Enzymatic Detection (SIEFED) Assay

SIEFED assay is a licensed method developed by Franck et al. [31] for the specific detection of animal origin MPO. MPO solution was made by diluting human MPO in 20 mM of phosphate buffer saline (at pH 7.4), 5 g/L BSA, and 0.1% Tween-20. Sample dilutions at final concentrations of 0.0125, 0.025, 0.05, 0.1, and 0.2 mg/mL were incubated for 10 min (at 37 °C) with MPO solution at a final concentration of 25 ng/mL. After incubation, the mixtures were loaded into the wells of a 96-well microtiter plate coated with rabbit polyclonal antibodies (3 µL/mL) against equine MPO and incubated for 2 h at 37 °C in darkness. After washing up the wells, the activity of the enzymes captured by the antibodies was measured by adding hydrogen peroxide (10 µM), NO_2_^−^ (10 mM) and Amplex^TM^ Red (40 µM). The oxidation of Amplex^TM^ Red into the fluorescent adduct resorufin was monitored for 30 min at 37 °C with Fluoroskan Ascent (Fisher Scientific, Asse, Belgium). Instead of sample dilutions only Milli-Q water was used in case of control.

### 2.7. Myeloperoxidase (MPO) Activity Using Classical Measurement

MPO solution was prepared as mentioned in Section 2.6. Sample dilutions at final concentrations of 0.0125, 0.025, 0.05, 0.1, and 0.2 mg/mL were incubated for 10 min (at 37 °C) with MPO solution at a final concentration of 25 ng/mL. After incubation, the mixture (100 µL) was immediately transferred into a 96-well microtiter plate. This was followed by the addition of 10 µL NO_2_^−^ (10 mM) and 100 µL of Amplex^TM^ Red and hydrogen peroxide mixture (at concentrations mentioned in Section 2.6). The oxidation of Amplex^TM^ Red into the fluorescent adduct resorufin was monitored for 30 min at 37 °C with Fluoroskan Ascent (Fisher Scientific, Asse, Belgium) immediately after addition of the revelation mixture. Instead of sample dilutions, only Milli-Q water was used in case of control.

### 2.8. Cellular Antioxidant Activity

Preparation of the neutrophil and phorbol 12-myristate 13-acetate (PMA) solutions were made according to Paul et al. [17]. The neutrophil response modulation activity of samples was analyzed using the protocol of Tsumbu et al. [16]. Neutrophil suspension (1 million cells/143 µL PBS) was loaded in wells of a 96-well microtiter plate and incubated for 10 min (at 37 °C in the dark) with phosphate buffer saline solution of samples at final concentrations of 0.0125, 0.025, 0.05, 0.1, and 0.2 mg/mL. After incubation, 25 µL calcium chloride (10 µM) and 20 µL L-012 (100 µM) was added in wells. The neutrophils were activated with 10 µL PMA (16 µM) immediately before monitoring the chemiluminesence response of neutrophils during 30 min at 37 °C using Fluoroskan Ascent (Fisher Scientific, Asse, Belgium). Instead of sample dilutions, only phosphate buffer saline was used in case of control.

### 2.9. Statistical Analyses

All the analyses were performed in triplicate. For protein quantification, the equation of a fitted line and R-square value were calculated using linear regression. The relationships between concentration and inhibition obtained for antioxidant assays were non-monotonic in nature. To address this, the locally estimated scatterpot smoothing (LOESS) regression technique was used to model the relationship [32]. Models were fitted using the R statistical software [33]. These models require a span parameter that defines the smoothing sensitivity of the local regressions. By visual inspection, a span parameter value of 0.4 was found to be suitable for all concentration and inhibition relationship curves. Concentrations with a predicted inhibition percentage of interest, such as IC_50_ (concentration at which 50% inhibition is reached), were found using the fitted models in combination with a numerical search routine.

## 3. Results

### 3.1. Protein Quantification

The calibration curve resulted in the following parameters: (1) equation of line: y = 0.3314x + 0.1503 (where x is the concentration of proteins); and (2) R-squared value: 0.9989. The optical density of samples and relative concentration of proteins (calculated using equation of line) are mentioned in Table 3. BSF-P extract solution (3 mg/mL) exhibits the highest and BSF-HP solution exhibits the lowest protein concentrations amongst the tested solutions using bicinchoninic acid assay. 

### 3.2. DPPH Assay

**Figure 1:**
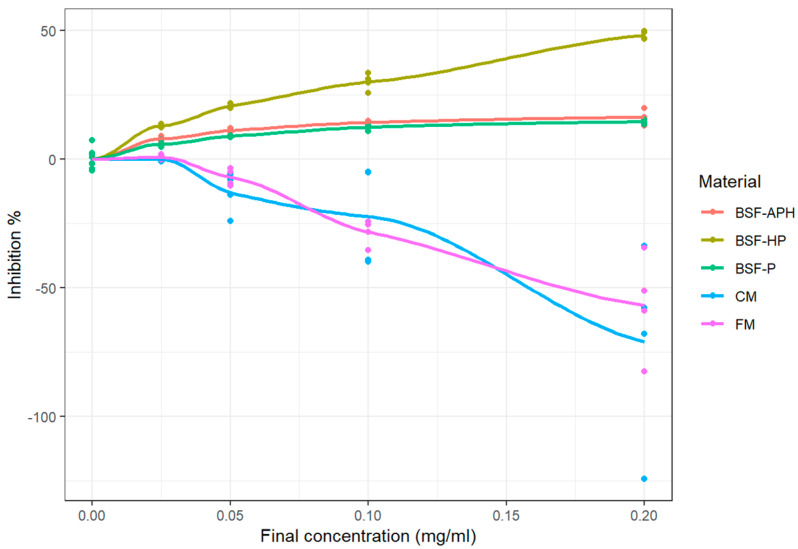
DPPH (2,2-diphenyl-1-picrylhydrazyl) radical scavenging activity of PureeX^TM^ (BSF-P), PureeX_pro_^TM^ (BSF-HP), ProteinAX_pro_^TM^ (BSF-APH), Chickenmeal (CM), and Fishmeal (FM) (n = 3).

The DPPH radical scavenging activity of all five samples after 30 min of incubation is indicated in Figure 1. The plot shows the measured values as well as fitted curves obtained from LOESS. CM exhibited pro-oxidant behavior at all tested concentrations. Whereas FM exhibited pro-oxidant behavior at four out of five tested concentrations. It was not possible to calculate IC_50_ for samples, because the samples either exhibited pro-oxidant activity or 50% inhibition was not achieved during the assay (see Table 4). The E_max_ (maximum inhibition achieved during the assay) of all the samples are also indicated in Table 5 and are in the following order: BSF-HP > BSF-APH > BSF-P > FM.

### 3.3. ABTS Assay

**Figure 2:**
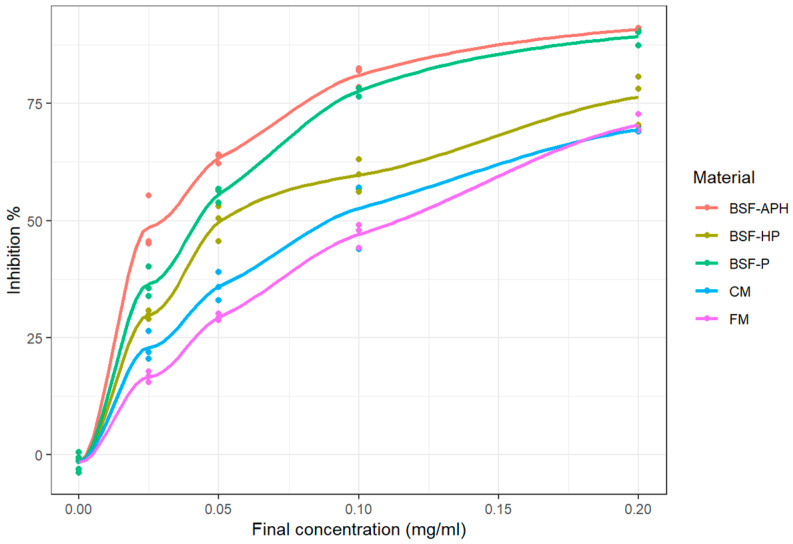
ABTS cation radical scavenging activity of PureeX^TM^ (BSF-P), PureeX_pro_^TM^ (BSF-HP), ProteinAX_pro_^TM^ (BSF-APH), chickenmeal (CM), and fishmeal (FM) (n = 3).

The ABTS cation radical scavenging activity of samples after 30 min of incubation is shown in Figure 2 (measured values as well as fitted curves obtained from LOESS). All the samples exhibited a similar inhibition pattern i.e., the percentage of inhibition increased as a function of increasing concentration. The IC_50_ of samples are mentioned in Table 4 and are in the following order: FM > CM > BSF-HP > BSF-P > BSF-APH. Lower IC_50_ reflects a higher ABTS cation radical scavenging activity. The E_max_ (maximum inhibition achieved during the assay) of all the samples are indicated in Table 5 and are in the following order: BSF-APH > BSF-P > BSF-HP > FM > CM.

### 3.4. Myeloperoxidase (MPO) Activity Using Specific Immunological Extraction Followed by Enzymatic Detection (SIEFED) Assay

**Figure 3:**
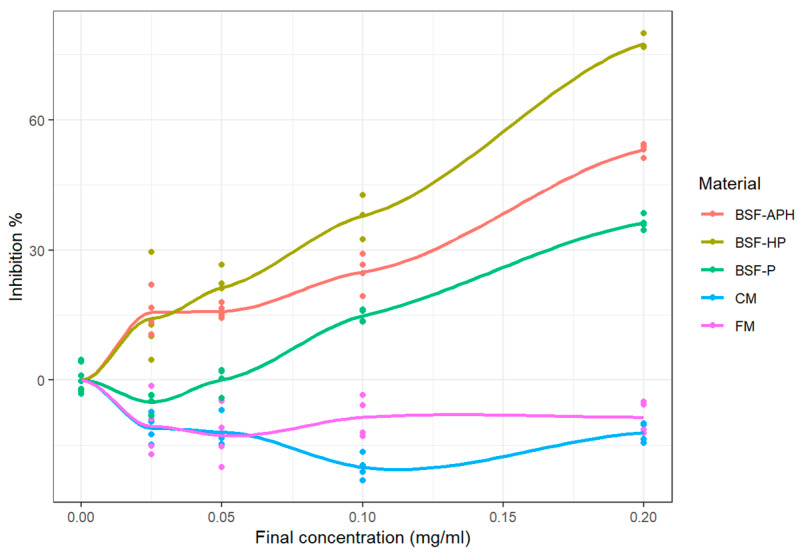
MPO response modulation activity of PureeX^TM^ (BSF-P), PureeX_pro_^TM^ (BSF-HP), ProteinAX_pro_^TM^ (BSF-APH), chickenmeal (CM), and fishmeal (FM) using specific immunological extraction followed by enzymatic detection (SIEFED) assay (n = 3).

The MPO response modulation activity of samples obtained using SIEFED assay is shown in Figure 3 (measured values as well as fitted curves obtained from LOESS). BSF-HP exhibited strong inhibition behavior, with >75% inhibition at 0.20 mg/mL concentration. The IC_50_ of samples are mentioned in Table 4 and are in the following order: BSF-APH > BSF-HP. The E_max_ of samples are shown in Table 5, and they are in the following order: BSF-HP > BSF-APH > BSF-P. FM and CM show pro-oxidant behavior at all tested concentrations. On the other hand, E_max_ for BSF-P was <50%.

### 3.5. Myeloperoxidase (MPO) Activity Using Classical Assay

**Figure 4:**
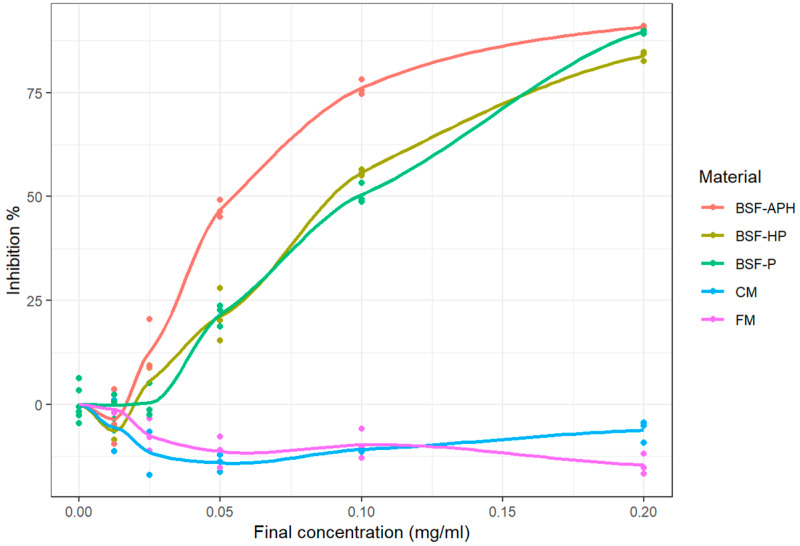
MPO response modulation activity of PureeX^TM^ (BSF-P), PureeX_pro_^TM^ (BSF-HP), ProteinAX_pro_^TM^ (BSF-APH), chickenmeal (CM), and fishmeal (FM) using classical measurement (n = 3).

The MPO response modulation activity of samples obtained using classical assay is indicated in Figure 4 (measured values as well as fitted curves obtained from LOESS). CM and FM exhibited pro-oxidant behavior at all tested concentrations. The E_max_ of all the samples tested are indicated in Table 5. BSF-APH, BSF-P, and BSF-HP exhibited E_max_ > 75%. The IC_50_ of samples are mentioned in Table 4 and are in the following order: BSF-P > BSF-HP > BSF-APH.

### 3.6. Cellular Antioxidant Activity

**Figure 5:**
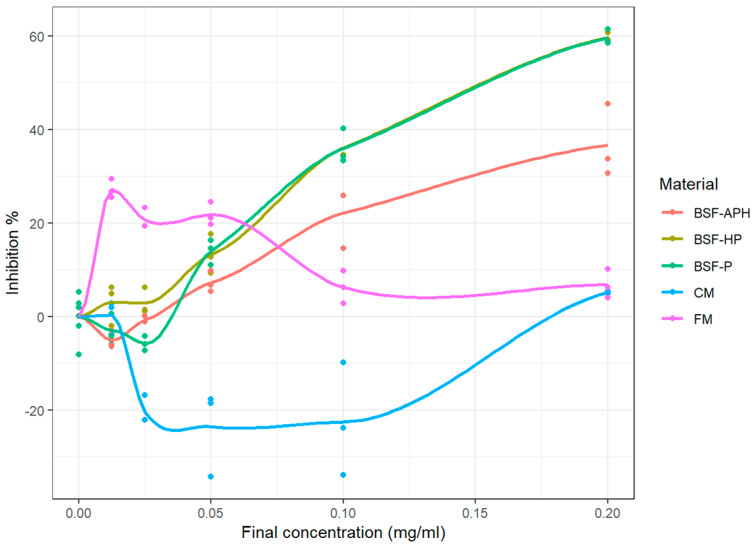
Neutrophil response modulation activity of PureeX^TM^ (BSF-P), PureeX_pro_^TM^ (BSF-HP), ProteinAX_pro_^TM^ (BSF-APH), chickenmeal (CM) and fishmeal (FM) (n = 3).

The neutrophil response modulation activity (measured values as well as fitted curves obtained from LOESS) and E_max_ of the samples are shown in Figure 5 and Table 5, respectively. All the tested samples exhibited E_max_ > 0%. BSF-APH, FM, and CM exhibited E_max_ < 40%. CM exhibited pro-oxidant behavior at 3 out of 5 tested concentrations. The IC_50_ of samples are mentioned in Table 4. BSF-P and BSF-HP have the same numerical IC_50_ values.

## 4. Discussion

### 4.1. Protein Quantification

The protein concentration of BSF-APH and four water-soluble extracts estimated using bicinchoninic acid assay are displayed in Table 3. For BSF-APH, 3 mg/mL solution resulted in protein concentration of 0.702 mg/mL, which translates into 0.235 mg proteins per gram of BSF-APH (or 23.5% proteins). According to the supplier, the average protein content of BSF-APH is 45.5% (see Table 2, analyzed using the Dumas method). Differences in protein content arise due to the method of analysis. Bicinchoninic acid assay is based on the detection of bonds specific to Cys, Trp, and Tyr. On the other hand, Dumas assay is based on the estimation of total organic nitrogen [34]. Therefore, protein content estimated using the Dumas method is always higher than that estimated using bichinchoninic acid assay. However, comparing the two protein estimation methods is not the goal of this study. Considering the amino acid pattern of black soldier fly proteins, FM and CM [35,36], it could be hypothesized that the protein content of four water-soluble extracts are in the following order: BSF-P > CM > FM > 45.5% > BSF-HP.

### 4.2. DPPH Radical Scavenging Activity

DPPH and ABTS assays are commonly used to analyze the antioxidant potential of food and feed products [17]. DPPH radical scavenging activity represents the ability of a sample to donate hydrogen atoms (referred as hydrogen atom transfer) or electrons (referred as single electron transfer) to stabilize free radicals [29]. DPPH assay IC_50_ and E_max_ for all tested samples are mentioned in Table 4 and Table 5, respectively. Post 30 min of incubation, all the tested samples exhibit E_max_ < 50% (with BSF-HP exhibiting highest E_max_). According to the supplier, BSF-HP is manufactured by the controlled hydrolysis of black soldier fly proteins and contains at least 24% of proteins <1000 Da (see Table 2). On the other hand, BSF-P and BSF-APH contain at least 6% and 98% proteins <1000 Da. The authors were not able to find any representative literature for the molecular weight distribution of FM and CM. However, according to the literature, FM and CM contain 2.2% and 1.1% free amino acid (of total proteins), respectively [37], which translates into FM and CM containing at least 2.2% and 1.1% proteins <1000 Da, respectively. Zou et al. [38] indicated that the capacity of proteinaceous materials to scavenge free radicals depends on the protein molecular weight distribution. Proteins with low molecular weight peptides could scavenge free radicals more efficiently. However, this does not explain the fact that BSF-APH contains a higher amount of proteins <1000 Da and still exhibits a lower inhibition of DPPH free radicals. The free radical scavenging activity of proteinaceous molecules is also influenced by the following. (1) Amino acid composition: hydrophobic amino acids (for e.g., Tyr, Phe, Pro, Ala, His and Leu) have superior radical scavenging activity in comparison to hydrophilic amino acids (2) Amino acid sequence: Peptides with an amphiphilic nature could enhance the radical scavenging activity of a sample [38,39,40]. Chemical analyses have indicated that Tyr exhibits antioxidant behavior via the hydrogen atom transfer mechanism. On the other hand, amino acids such as Cys, Trp, and His exhibit antioxidant behavior via the single electron transfer mechanism [41].

FM and CM exhibit pro-oxidant behavior at most of the tested concentrations (see Figure 1). This behavior mainly arises from the thermal processing. For both FM and CM, thermal processing commonly involves heating the raw product at high temperatures for 15 to 20 min [42,43]. In Norway, during fishmeal production, wild caught fish are subjected to heating at temperatures ≥70 °C for time ≥20 min in order to achieve 100 log_10_ reductions of *Enterobacteriaceae* and *Salmonella* counts [44]. Such strict thermal processing conditions may result in the oxidation of fats and proteins. Fishmeal contains lipids rich in polyunsaturated fatty acids that are more susceptible to thermal oxidation [45]. Antioxidants are commonly added in fishmeal to prevent the oxidation of polyunsaturated fatty acids (also visible in Table 1). The heat-induced oxidation of amino acids leads to the development of a wide range of oxidation products [45,46]. The pro-oxidant behavior of amino acid oxidation by-products is already known. They can result in a wide range of health conditions in animal body [47]. According to the supplier, all the black soldier fly protein derivatives used in this study were thermally processed at temperatures <100 °C for time <1.5 min. The supplier also indicated that these thermal processing time–temperature combinations were adopted to ensure minimum damage to nutrients (proteins and fat) and the adequate inactivation of pathogenic microbiota. This implies that the pro-oxidant behavior of FM and CM arises mainly due to the stringent production method.

In a recent study [27], researchers made BSF protein hydrolysate using a bromelain enzyme. Bromelain-derived protein hydrolysate was also tested for DPPH radical scavenging activity, which resulted in the IC_50_ of 8.4 mg/mL. The DPPH radical scavenging activity of this bromelain-derived protein hydrolysate was much lower when compared to the activity of products such as BSF-HP (IC_50_ 0.18 mg/mL after 60 min of incubation). The higher activity of BSF-HP used in this study could arise from compositional attributes (as previously discussed in this section) and the quality of the raw material itself. Protix is reportedly producing insect proteins in GMP+ and SecureFeed certified facilities, under HACCP conditions [7].

### 4.3. ABTS Cation Radical Scavening Activity

ABTS cation radical scavenging denotes the ability of a sample to donate electron and stabilize free radicals [17]. The ABTS assay IC_50_ of all samples are indicated in Table 4. They are in the following order: FM > CM > BSF-HP > BSF-P > BSF-APH. The higher the IC_50_, the lower the antioxidant activity. In this assay, even FM and CM exhibit antioxidant activity. It appears that FM and CM extracts may be efficient where free radical(s) could be stabilized using a single electron transfer mechanism. However, they still exhibit lower scavenging activity in comparison to BSF derivatives. 

BSF-APH has at least 98% proteins <1000 Da (the lowest protein molecular weight amongst all tested samples) and exhibited the lowest ABTS IC_50_. The dependence of radical scavenging activity on protein molecular weight is already explained in Section 4.2. According to the supplier, BSF-P and BSF-HP have the same amino acid composition. However, due to protein hydrolysis (hydrolysis details were not disclosed by the manufacturer), the amount of proteins <1000 Da is higher in BSF-HP than in BSF-P. It is therefore somewhat surprising that the BSF-P IC_50_ value was slightly lower in comparison to BSF-HP. This could be explained by the mechanism of hydrolysis. Enzymatic hydrolysis is achieved through exo- and endopeptidase. Exopeptidase cleaves the terminal peptide bond; on the other hand, endopeptidase cleaves the non-terminal peptide bond [48]. In both cases, the sequence of amino acids is altered. The radical scavenging ability of the resulting peptides via single electron transfer is also dependent on the amphiphilic nature of proteinaceous molecules [38]. It is possible that the peptides in BSF-HP are less amphiphilic in nature, which results in the lower ABTS cation radical scavenging activity of BSF-HP compared to BSF-P.

Zhu et al. [26] developed BSF protein hydrolysate using a wide range of commercial enzymes. The hydrolysates were further fractionated into the following groups using ultrafiltration: group 1 (<3000 Da), group 2 (3000 to 10,000 Da), and group 3 (>10,000 Da). The activity of these hydrolyzed fractions was also investigated for ABTS cation radical scavenging activity. Ascorbic acid was used as the reference molecule in this study. Interestingly, the best performing fraction and ascorbic acid were able to inhibit 85.7% and 92.1% of ABTS cation radicals at 0.05 mg/mL concentration, respectively. In our study, BSF-P and BSF-APH exhibit ABTS cation radical scavenging E_max_ of 89% and 91%, respectively (at 0.2 mg/mL). This indicates that fractioning BSF-P and BSF-APH could result in fractions that may have very strong antioxidant potential.

### 4.4. Neutrophil Response Modulation Activity

**Figure 6:**
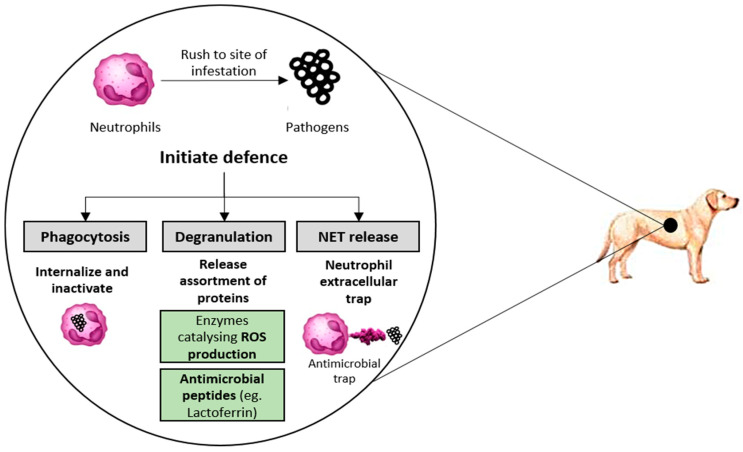
Defense mechanism of neutrophils. Source: Adapted from Tsumbu et al. [16], Paul et al. [17], and Perobelli et al. [44].

The strong free radical scavenging activities of BSF derivatives are evident from Section 4.2 and Section 4.3. Furthermore, all the samples were also tested for neutrophil response modulation activity. Neutrophils are white blood cells present in the animal body (including humans, pets, fishes, poultry, and swine). They are involved in the primary defense against pathogens [16]. When pathogenic microbes enter the animal body, neutrophils rush to the site of infestation and initiate defense via the mechanism indicated in Figure 6. During degranulation, neutrophils release a wide range of oxidative enzymes including myeloperoxidase, which results in the activation of nicotinamide adenine dinucleotide phosphate (NADPH) oxidase. NADPH oxidase is responsible for the production of superoxide anion and by-products (e.g., hydrogen peroxide) [16,17]. Superoxide anion can further react with the nitric oxide radical to produce peroxynitrite. This process also generates a hydroxyl radical (by the reaction of hydrogen peroxide with metal ion) [49,50]. This battery of oxidative reactions is crucial to the defense of the host animal. However, these ROS generated during host defense can react with enzymes, proteins, lipids, etc., of body cells and result in the development of various health conditions (for e.g., cellular aging, cancer, etc.) [18]. The neutrophil assay conducted in this research determines the ability of proteinaceous molecules to scavenge ROS produced as a result of neutrophil activity. PMA was used to activate protein kinase C present in neutrophils, which results in the production of NADPH oxidase responsible for catalyzing ROS production. ROS production in the system is coupled with lucigenin-amplified chemiluminescence. The ability of a proteinaceous sample to scavenge ROS (particularly superoxide anion) is marked by a decreased chemiluminescence [51].

To the author’s knowledge, this is the first analysis of the *in vitro* neutrophil response modulation activity of BSF derivatives. CM exhibited pro-oxidant behavior at 3 out of 5 tested concentrations and had an E_max_ of only 5% at 0.2 mg/mL (see Figure 5 and Table 5). CM is commonly used in pet food preparations [52]. However, the outcomes of the current study indicate that CM inclusion offers little or no benefits relating to scavenging the ROS produced by activated neutrophils. Moreover, CM inclusion could even result in inflammatory damage to host cells. The repetitive inflammatory damage of canine or feline cells could translate into conditions such as accelerated aging, slow cognitive function, etc. [14].

On the other hand, FM exhibits mild antioxidant behavior in this assay, with an E_max_ of 22% (see Table 5). At 0.2 mg/mL, FM exhibits inhibition of 5%. Aquaculture rearing media (i.e., water) offer a continuous buffer of pathogenic bacteria. Therefore, aquaculture organisms are at the constant risk of pathogenic bacterial invasions [53]. This results in a wide range of health conditions, including reduced immunity, aging, etc. [15]. Our research highlights the inadequacy of FM to suppress the inflammatory damage from repetitive neutrophil activity. This often translates into incremental cost occurring as a result of antibiotics and nutritional supplement usage. BSF-P used in the study is also the raw material to produce BSF proteinmeal. EU legislations already permit the use of insect proteinmeal in aquaculture diets. BSF-P exhibits E_max_ and IC_50_ of 59.57% and 0.15 mg/mL, respectively (see Table 5). Additionally, the supplier uses low drying temperature to convert BSF-P into BSF proteinmeal. This implies that BSF proteinmeal will have activity similar to BSF-P. Therefore, BSF-P-derived proteinmeal could also be effective in preventing the inflammatory damage resulting from neutrophil activity in the fish body. Moreover, BSF-HP also exhibits neutrophil response modulation activity comparable to BSF-P (see Table 4).

Therefore, it is possible that BSF derivatives (particularly BSF-P and BS-HP used in this study) could offer a natural and sustainable solution to suppress oxidative damage resulting from pathogenic invasion. The use of these ingredients may even help the aquaculture industry to improve the immune health of fishes.

### 4.5. MPO Response Modulation Activity (SIEFED and Classical Assay)

The general mechanism of neutrophil response is indicated in Figure 6. The neutrophil extracellular trap contains several molecules required to inactivate pathogenic microbes. The MPO enzyme present in neutrophil extracellular trap can produce hypochlorous acid from hydrogen peroxide and chloride ion. Additionally, MPO is capable of oxidizing tyrosine into the tyrosyl free radical. Both products of MPO oxidation (hypochlorous acid and tyrosyl free radical) are crucial to inactivate pathogens. Again, the repetitive interaction of these molecules with animal cells results in inflammatory damage [16,31]. In an animal body, MPO-Fe (III) (active form) reacts with hydrogen peroxide to form oxoferryl π cation radical (CpI form). CpI form converts back into MPO-Fe (III) coupled with chloride ion, transforming into hypochlorous acid. However, in the present experiment, back reduction of the Cp I form to MPO-Fe (III) was achieved in 2 stages. First, there was the reduction of CpI to MPO-Fe (IV) = O via electron transfer through nitrite ions. Then, electron provisioning was done (via Amplex^TM^ Red oxidation to resorufin reaction), which converts MPO-Fe (IV) = O to MPO-Fe(III) form [17,31,51]. Proteinaceous molecules could prevent the oxidative damage resulting from MPO either by directly reacting with the CpI form and terminating the halogenation or by donating hydrogen (hydrogen atom transfer) to ROS produced as a consequence of MPO activity [16]. The MPO response modulation activity was analyzed using the classical and SIEFED assay. The classical assay measures the ability of a sample to complex with CpI form and stabilize ROS. Whereas in SIEFED assay, MPO is bound to rabbit polyclonal antibodies (and the rest of the compounds are washed away), so it purely measures the ability of samples to complex with the CpI form [31].

As with neutrophil response modulation activity, the MPO response modulation activity of BSF derivatives is also being reported for the first time. FM and CM exhibit pro-oxidant behavior in both the assays (see Figure 3 and Figure 4). The presence of oxidative reaction products in FM and CM (because of the production process) that are capable of initiating pro-oxidative response have been already discussed in Section 4.2. Detailed *in vitro* investigations realized during this study indicate that the inclusion of FM and CM in animal diets may result in inflammatory damage.

In the classical assay, BSF derivatives exhibit strong antioxidant potential, with IC_50_ in following order: BSF-P > BSF-HP > BSF-APH. BSF-APH show strong antioxidant potential in the classical assay (see Table 4), whereas, for SIEFED assay, IC_50_ were in the following order: BSF-APH > BSF-HP. In the SIEFED assay, BSF-P did not reach 50% inhibition (even at the highest concentration tested). Thus, while BSF-P and BSF-APH are more effective in stabilizing ROS, BSF-HP has higher efficacy in complexing with the CpI form of MPO. These observations indicate that BSF derivatives could be used in pet food and aquaculture formulations to effectively suppress inflammatory damages resulting from MPO activity.

Free amino acids are directly absorbed from the animal intestine [54]. Whereas, the intestinal absorption of peptides takes place by one of the following mechanisms: (1) transfer and uptake of di- and tri-peptides by PepT1; (2) paracellular transport of water-soluble and low molecular weight peptides via intercellular junctions; and (3) uptake of short and intermediate peptides by transcytosis [55], indicating that the water-soluble extracts used in this study will pass the intestinal membrane with minimum alterations. Due to this reason, BSF-APH, with high water solubility (>95%) and the majority of proteins below 1000 Da (>98%), could be a very interesting candidate for inclusion in pet and fish diets to promote animal health. In the future, it could be of interest to analyze the effect of feed processing treatments on the *in vitro* bioactivity of BSF protein derivatives.

BSF protein derivatives used in this study offer an antioxidative advantage over FM and CM. However, the animal body is a complex system with several biochemical processes taking place simultaneously. Additionally, several processes interact with each other, resulting in an adapted response [56]. It is possible that BSF protein derivatives show an altered response in the animal body. Therefore, in the future, it could also be interesting to investigate the activity of BSF protein derivatives using *in vivo* animal feeding trials.

## 5. Conclusions

In this study, the *in vitro* antioxidant activity of commercial black soldier fly proteins and protein hydrolysates was analyzed using radical scavenging models (DPPH and ABTS assays), enzymatic models involving myeloperoxidases activity modulation (classical and SIEFED assays), and a cellular model involving neutrophil response modulation. Commercial fishmeal and chickenmeal were used as industrial benchmarks. The outcomes of the present study reveal that fishmeal and chickenmeal offer little to no advantage in terms of suppressing the oxidative damage occurring as a result of neutrophil response and myeloperoxidase activity. Moreover, fishmeal and chickenmeal also exhibit pro-oxidant behavior in some of the models used in this study. Results indicate that black soldier fly proteins and protein hydrolysate could be effective in protecting against the cellular damage resulting from host neutrophil and myeloperoxidase response. Therefore, the black soldier fly derivatives used in this study show advantages over chickenmeal and fishmeal for inclusion in pet food and aquaculture feed formulations. In the future, it could be interesting to validate the fundamental *in vitro* knowledge developed during this study using *in vivo* animal models.

## Figures and Tables

**Table 1:** Chemical composition of chickenmeal and fishmeal (as in basis, provided by supplier).

Nutrients	Chickenmeal	Fishmeal
Moisture (g/kg)	60.0	100.0
Crude protein (g/kg)	700.0	710.0
Crude fat (g/kg)	120.0	120.0
Added antioxidant	No	Yes (E324 *)
Form	Powder	

* E324: Ethoxyquin.

**Table 2:** Chemical composition of black soldier fly larvae (BSF) protein derivatives (as in basis, provided by supplier).

Nutrients	BSF-P ^1^	BSF-HP ^2^	BSF-APH ^3^
Moisture (g/kg)	700.0 ^a^	700.0 ^a^	55 ^a^
Crude protein (g/kg)	120 ^a^	120 ^a^	455 ^a^
Crude fat (g/kg)	122.5 ^a^	122.5 ^a^	35 ^a^
Added antioxidant	No	No	No
% of total proteins <1000 Da	>6	>24	>98
Form	Frozen minced meat		Powder

^1^ BSF-P: PureeX^TM^; ^2^ BSF-HP: PureeX_pro_^TM^; ^3^ BSF-APH: ProteinAX_pro_^TM^; ^a^ Mean values based on the range proposed by supplier.

**Table 3:** Protein quantification using bicinchoninic acid assay.

Product	Product Used for Testing in all the Assays	Mean Optical Density	Protein Concentration (mg/mL)
BSF-P ^1^	Water-soluble extract	0.486	1.013
BSF-HP ^2^	Water-soluble extract	0.365	0.648
BSF-APH ^3^	Product as provided by supplier	0.383	0.702
FM ^4^	Water-soluble extract	0.425	0.829
CM ^5^	Water-soluble extract	0.481	0.998

^1^ BSF-P: PureeX^TM^; ^2^ BSF-HP: PureeX_pro_^TM^; ^3^ BSF-APH: ProteinAX_pro_^TM^; ^4^ FM: Fishmeal; ^5^ CM: Chickenmeal.

**Table 4:** Antioxidant activity IC_50_ (mg/mL) of samples obtained using different assays.

Assay	BSF-P ^1^	BSF-HP ^2^	BSF-APH ^3^	FM ^4^	CM ^5^
DPPH	NE ^c^	NE ^c^	NE ^c^	NE ^c^	PO ^d^
ABTS	0.04	0.05	0.03	0.11	0.09
MPO ^a^ SIEFED	NE ^c^	0.14	0.18	PO ^d^	PO ^d^
MPO ^a^ Classical	0.10	0.09	0.05	PO ^d^	PO ^d^
CAA ^b^	0.15	0.15	NE ^c^	NE ^c^	NE ^c^

^1^ BSF-P: PureeX^TM^; ^2^ BSF-HP: PureeX_pro_^TM^; ^3^ BSF-APH: ProteinAX_pro_^TM^; ^4^ FM: Fishmeal; ^5^CM: Chickenmeal; ^a^ MPO: Myeloperoxidase; ^b^ CAA: Cellular antioxidant activity using neutrophil model; ^c^ NE: Not estimated because 50% inhibition was not achieved in tested concentrations; ^d^ PO: Not estimated because sample exhibited pro-oxidant activity on tested concentrations.

**Table 5:** Antioxidant activity E_max_ (% inhibition) of samples obtained using different assays. ABTS: 2,2’-azino-bis(3-ethylbenzothiazoline-6-sulfonic acid.

Assay	Parameter	BSF-P ^1^	BSF-HP ^2^	BSF-APH ^3^	FM ^4^	CM ^5^
DPPH	Emax (%)	14.52	48.09	16.26	0.75	PO ^c^
	C * (mg/mL)	0.20	0.20	0.20	0.03	-
ABTS	Emax (%)	89.33	76.32	90.81	70.40	69.39
	C * (mg/mL)	0.20	0.20	0.20	0.20	0.20
MPO ^a^ SIEFED	Emax (%)	36.23	77.58	53.08	PO ^c^	PO ^c^
	C * (mg/mL)	0.20	0.20	0.20	-	-
MPO ^a^ Classical	Emax (%)	89.66	83.82	90.86	PO ^c^	PO ^c^
	C * (mg/mL)	0.20	0.20	0.20	-	-
CAA ^b^	Emax (%)	59.57	59.64	36.62	21.81	5.08
	C * (mg/mL)	0.20	0.20	0.20	0.05	0.20

* C: Concentration at which E_max_ is achieved; ^1^ BSF-P: PureeX^TM^; ^2^ BSF-HP: PureeX_pro_^TM^; ^3^ BSF-APH: ProteinAX_pro_^TM^; ^4^ FM: Fishmeal; ^5^ CM: Chickenmeal; ^a^ MPO: Myeloperoxidase; ^b^ CAA: Cellular antioxidant activity using neutrophil model; ^c^ PO: Not estimated because sample exhibited pro-oxidant activity on tested concentrations.
